# Environmental factors and awareness of colorectal cancer in people at familial risk^*^

**DOI:** 10.1590/1518-8345.3082.3195

**Published:** 2019-10-14

**Authors:** Luis Arturo Pacheco-Pérez, Karla Judith Ruíz-González, Aldo César Gómez de-la-Torre-Gómez, Milton Carlos Guevara-Valtier, Linda Azucena Rodríguez-Puente, Juana Mercedes Gutiérrez-Valverde

**Affiliations:** 1 Universidad de Sonora. Departamento de Ciencias de la Salud, Ciudad Obregón, Sonora, México.; 2 Bolsista da CONACYT, México.; 3 Hospital General "Dr Salvador Zubirán Anchondo". Supervisión de Enfermería, Chihuahua, Chihuahua, México.; 4 Instituto Mexicano del Seguro Social, Atención Integral a la Salud en el Primer Nivel, Ciudad de México, México.; 5 Universidad Autónoma de Nuevo León, Facultad de Enfermería, Monterrey, Nuevo León, México.; 6 Universidad Autónoma de Coahuila, Facultad de Enfermería, Saltillo, Coahuila, México.

**Keywords:** Colorectal Neoplasms, Risk Factors, Awareness, Primary Prevention, Early Detection of Cancer, Heredity, Neoplasias Colorretais, Fatores de Risco, Conscientização, Prevenção Primária, Detecção Precoce de Câncer, Hereditariedade, Neoplasias Colorrectales, Factores de Riesgo, Concienciación, Prevención Primaria, Detección Precóz del Cáncer, Herencia

## Abstract

**Objective:**

to identify the association between environmental risk factors and awareness of colorectal cancer in people at familial risk.

**Method:**

cross-sectional correlational study, with a sample consisted of people who met at least one of the Revised Bethesda criteria, and 80 participants were included in this study. A sociodemographic data record, the AUDIT Test for alcohol use, the Fagerström Test for tobacco smoking, the Estimation and Frequency of Food Intake scale, and the Cancer Awareness Measure questionnaire to assess the colorectal cancer awareness were used. Body mass index was calculated, and descriptive statistics and the Pearson’s Correlation Coefficient were used to estimate the association.

**Results:**

female sex predominated, with an average age of 37.8 years, almost half of the participants were overweight, 45% showed symptoms of alcohol dependence, half of the sample showed an association between hereditary factors and the development of colorectal cancer, and less than half of them were aware of cancer prevention programs.

**Conclusion:**

there is little information on the main environmental risk factors, signs and symptoms of colorectal cancer, and no significant association was found between these and colorectal cancer awareness.

## Abstract (pt)

**Objetivo:**

identificar a associação entre os fatores de risco ambientais e a conscientização sobre o câncer colorretal em pessoas com risco familiar.

**Método:**

estudo correlacional de corte transversal, cuja amostra foi constituída por pessoas que cumpriam pelo menos um dos critérios de Bethesda Revisados, sendo 80 participantes incluídos no estudo. Foram utilizados uma ficha com dados sociodemográficos, o Teste AUDIT para o consumo de álcool, o Teste de Fagerström para o tabagismo, a escala de Estimativa e Consumo de Alimentos, e o questionário *Cancer Awareness Measure* para avaliar a conscientização sobre o câncer colorretal. O índice de massa corporal foi calculado, e estatísticas descritivas e o Coeficiente de Correlação de Pearson foram usados para estimar a associação.

**Resultados:**

o sexo feminino foi o mais prevalente, com média de idade de 37,8 anos, quase metade dos participantes apresentava sobrepeso, 45% apresentavam sintomas de dependência de álcool, metade da amostra teve o fator hereditário relacionado com o desenvolvimento de câncer colorretal, e menos da metade conhecia programas preventivos deste câncer.

**Conclusão:**

há pouca informação sobre os principais fatores de risco ambientais, sinais e sintomas do câncer colorretal, e nenhuma associação significativa foi encontrada entre eles e conscientização sobre o câncer colorretal.

## Abstract (es)

**Objetivo:**

identificar la asociación entre factores de riesgo ambientales y la conciencia sobre el cáncer colorrectal en personas con riesgo familiar.

**Método:**

estudio transversal correlacional; la muestra estuvo constituida por personas que cumplieran al menos uno de los criterios de Bethesda Revisados; se incluyeron 80 participantes en el estudio; se empleó una cédula de datos sociodemográficos, el Test AUDIT para el consumo de alcohol, Test de Fagerström para el consumo de tabaco, escala Estimación y Consumo de Alimento y el cuestionario Cancer Awareness Measure para la conciencia sobre el cáncer colorrectal, además se calculó el índice de masa corporal; se obtuvo estadística descriptiva y el Coeficiente de Correlación de Pearson para la asociación.

**Resultados:**

predominó el sexo femenino, con una media de edad de 37,8 años; casi la mitad de los participantes presentó sobrepeso, un 45% mostró síntomas de dependencia al alcohol, la mitad de la muestra relaciona el factor hereditario con el desarrollo de cáncer colorrectal y menos de la mitad conoce programas preventivos sobre el mismo.

**Conclusión:**

existe poca información sobre los principales factores de riesgo ambientales, signos y síntomas del cáncer colorrectal; no se encontró una asociación significativa entre estos y la conciencia sobre el cáncer colorrectal.

## Introduction

Colorectal cancer (CRC) is one of the most common types of cancer, the third most incident cancer and the fourth leading cause of deaths from malignant tumors, accounting for approximately 774,000 deaths in 2015 worldwide^1^. In the Americas region, CRC is the fourth most common type of cancer, with 240,000 new cases each year, accounting for approximately 112,000 deaths, and it is predicted that by 2030 its incidence rate will increase by 60%^2^.

There are multiple environmental factors associated with CRC diagnosis and different studies indicate that obesity, alcohol use, tobacco smoking, intake of red and processed meat are related to CRC, while factors such as physical activity and maintaining an appropriate weight can reduce the risk of developing it^3-7^. It has been identified that CRC is also associated with hereditary factors, since having at least one first-degree relative diagnosed with CRC increases the risk of developing the disease^8^. Moreover, it has been found that people over 50 years are more likely to develop CRC^8-10^.

It is important to emphasize that most cases of CRC due to sporadic causes or environmental factors. However, 10-30% of cases of CRC occur when there is a relative with the same diagnosis^11^. Lynch syndrome (LS) is the most frequent cause of hereditary CRC and is attributed to up to 5% of cases^11-15^. In addition, alcohol use, tobacco smoking, intake of red and processed meat, as well as obesity, are associated with an early CRC diagnosis in people at risk for LS^15-18^.

There are several methods to diagnose LS, however, strategies such as prevention and awareness of the risk of developing the disease are equally effective and have lower costs. CRC risk awareness refers to knowledge on the warning signs, risk factors, delay in seeking medical help in case of having signs or symptoms, high-risk age, risk of developing CRC throughout life, knowledge on timely detection programs and confidence in detecting symptoms^19^.

Several researches have aimed to study CRC awareness worldwide and they agree that there is little knowledge about the risk factors, signs and symptoms of this disease^20-22^. There is a need to implement strategies to raise CRC awareness and identify possible cases of LS to prevent the development of the disease at an early age, since cancer can develop before the age of 30 in the case of having this health condition.

It is important to point out that CRC prevention is a development field little explored by the nursing professional who works in different contexts, due to the limited evidence found in Latin America on studies led by nurses that include the genetic and hereditary component of CRC, such as in the case of LS.

The nursing professional represents an essential role in disease prevention and promotion of awareness of the risk of developing CRC, since these health professionals are those with a closer contact with patients and their families in the community and hospital context. In addition, their training in the health education field empowers them to formulate and propose interventions designed to stimulate a culture of health promotion and disease prevention with the aim of modifying health risk behaviors.

Based on the above, the purpose of this study was to determine the relationship between environmental factors (alcohol use, tobacco smoking, food intake, body mass index [BMI]) and CRC risk awareness in people at familial risk.

## Method

Study with a cross-sectional descriptive design, developed in two public health institutions in the city of Chihuahua, Mexico, whose data collection was carried out between August and September 2016. The study population consisted of people at familial risk, of which 167 were patients diagnosed with CRC who were under treatment and diagnosed before the age of 50. Once the list of patients diagnosed with CRC was available, these people were contacted by telephone so that they could provide the data of a relative who wished to participate, since the study was composed of people at risk of developing CRC. Each patient provided information about a family member who was contacted by telephone and to whom the purpose of the study was explained.

The sample was calculated using the nQuery Advisor^®^ program for multiple regression, with a significance level of 0.05, bilateral alternative hypothesis, effect size of 0.7 and a power of 80%. The final sample size was composed of 68 individuals, considering rejection and abandonment rates of 16%, according to a similar study^23^, from a total of 80 participants, and a simple random sampling was performed using the random function of the Microsoft Excel program^®^.

People at familial risk, aged between 18 and 60 years and who met at least one of the Revised Bethesda criteria^14^ were included, and those participants who were under any treatment to stop using alcohol, tobacco smoking or lose weight during the study period were excluded to reduce bias.

A sociodemographic data record and four instruments were used and anthropometric measures of weight and height were performed to calculate the BMI.

Alcohol use. It was used an instrument validated with a population from Mexico: The Alcohol Use Disorders Identification Test^24^ AUDIT, which consists of 10 items assigned in the following domains: hazardous alcohol use (items 1, 2 and 3), dependence symptoms (items 4, 5 and 6), and harmful alcohol use (items 7 to 10). In this questionnaire, dependence scores of 0 to 7 indicates low-risk; 8 to 15 indicates moderate risk or hazardous level, and 16 to 40 a high-risk or harmful level of alcohol dependence. The instrument showed a Cronbach’s alpha of 0.83.

Tobacco smoking. The Fagerström Test that has been validated to Spanish was used to assess nicotine dependence^25^. The questionnaire consists of six items, with dependence scores of 0 to 10, where a score of six points or more indicates a high level of tobacco dependence. This instrument showed a Cronbach’s alpha of 0.84.

Food Intake. The Estimation and Frequency of Food Intake Scale^26^ (EFI) was used, which consists of 23 items with Likert-type response options for estimation (very unhealthy, little unhealthy, regularly healthy, healthy and very healthy) and frequency of intake (daily, 2-3 times a week, once a week, once a month and never), and the interpretation of the instrument is descriptive. The instrument showed an internal consistency of 0.92 for the estimation and 0.90 for the frequency of food intake, and it was developed and validated in Spanish with a population from Mexico. The EFI showed a Cronbach’s alpha of 0.87 in this study.

CRC Awareness. The *Bowel Cancer Awareness Measure* ^27-28^ (CAM) was used, which consists of 8 items, where items 1, 3 and 5 represent open questions and the rest are multiple choice questions. It evaluates the knowledge of the signs and symptoms: constipation, diarrhea, presence of blood in the stool, unexplained loss of weight and pain, risk factors such as obesity, high red meat and low fiber diet, alcohol use and tobacco smoking, hereditary risk, diabetes and age. It also evaluates the knowledge about prevention programs and confidence in identifying the CRC symptoms, and its interpretation is descriptive. The instrument showed a Cronbach’s alpha of 0.81. During this study, no CAM validated to Spanish was found, but the process of implementing this instrument is described in another similar study conducted with a population from Mexico^29^.

BMI. Anthropometric measurements of body weight and height were performed^30^. A *Teraillon* ^®^ portable digital scale was used, and whose accuracy was previously checked by comparing the weights of five different objects estimated using other scales. A *Zaude* ^®^ wall-mounted stadiometer with 1mm precision was used to measure height, and two measurements were performed, whose values were averaged, registered and used to calculate the BMI of each individual. BMI values > 25kg/m^2^ was considered as overweight, and BMI > 30kg/m^2^as obesity^31^.

The Committee of Ethics in Research approved the study to ensure the integrity of participants and the Research Committee approved the study to ensure that methodology and analysis were appropriate, both committees attached to the Faculty of Nursing of the Autonomous University of Nuevo León (protocol: FAEN-D-1184). This is so because this study derived from a counseling intervention project for people at risk for CRC, in order to promote awareness of the disease. Prior to data collection, participants were informed of their right to renounce and withdraw their information at any time, as well as the proper management of their data and after that, each participant signed an informed consent form.

Data was analyzed using SPSS^®^ version 21 for Windows. Frequencies, measures of central tendency and dispersion were obtained; the Kolmogorov-Smirnov Goodness of Fit Test was performed with Lilliefors correction to check the normality of the data, since the variables of interest showed normality, the Pearson’s Correlation Coefficient test was used with the indexes and summations of each variable, in addition to the contingency coefficient for qualitative variables. A 95% confidence level was used, and *p* < 0.05 was considered statistically significant.

## Results

In the study, female sex predominated (55%), the majority of participants were married (62.5%), the average age was 36.8 years (SD = 10.72); the alcohol use index showed an average of 18.56 (SD = 15.04) and 45% had dependence symptoms. The average of tobacco dependence was 3.50 (SD = 11.88) and 90% of participants did not smoke. The mean BMI was 28.21 (SD = 6.44) and 41.2% were overweight (Table 1).

**Table 1:** Sociodemographic characteristics, BMI*, alcohol use and tobacco smoking of the 80 participants. Chihuahua, Chih, Mexico, 2016

Variable	f^†^	%
Gender		
Female		
Male	44	55
Marital status	36	45
Married		
Single	50	62,5
Body Mass Index (Kg/m^2^)	30	37,5
Underweight (< 18.5)		
Healthy weight (18.5 – 24.9)	1	1,2
Overweight (25 – 29.9)	22	27,5
Obesity Class 1 (≥ 30 – 34.9)	33	41,2
Obesity Class 2 (≥ 35 – 39.9)	17	21,2
Obesity Class 3 (≥ 40)	3	3,7
Alcohol use	4	5
Do not use alcohol		
Hazardous alcohol use	24	30
Dependence symptoms	9	11,2
Harmful alcohol use	36	45
Tobacco smoking	11	13,7
Do not smoke		
Dependence (< 6 points)	72	90
High degree of dependence (> 6 points)	8	10

*BMI = Body Mass Index; ^†^f = frequency

In relation to food intake, 23.8% indicated that *carne en su jugo* is healthy and 18.8% of participants eat it once a week; while almost one third of participants consume fat meat and beef tacos once a week (Table 2).

**Table 2:** Estimation and frequency of food intake of the 80 participants. Chihuahua, Chih, Mexico, 2016

Variable	Estimation				Frequency			
Food intake	Very healthy		Healthy		2-3 times a week		Once a week	
	f*	%	f*	%	f*	%	f*	%
Bacon	2	2,5	2	2,5	3	3,8	13	16,3
Fat meat	2	2,5	9	11,3	10	12,5	31	31,8
Beef burgers	-	-	2	2,5	5	6,3	11	13,8
Mexican tacos of beef	-	-	2	2,5	3	3,8	26	32,5
Mexican tacos of barbecue or ground beef	-	-	1	1,3	3	3,8	9	11,3
*Carne en su jugo*	3	3,8	19	23,8	5	6,3	15	18,8
Sausage	-	-	6	7,5	10	12,5	27	33,8
Hot dog	2	2,5	1	1,3	5	6,3	22	27,5
Stewed beef	1	1,3	11	13,8	11	13,8	16	20
Mexican tacos of pork	1	1,3	4	5	4	5	19	23,8

*f = frequency

According to the results on CRC awareness, it was observed that bleeding in digestive tract is the most well-known symptom (58.8%) and less than half of the participants had the CRC diagnosis associated with age (46.3%). In total, 50% of individuals report that having a close family member diagnosed with CRC is a risk factor for developing this disease, 45% of people are aware of a CRC prevention program and only 13.8% of the participants reported having confidence in detecting CRC signs and symptoms in case of presenting it (Table 3).

**Table 3:** CRC* awareness of the 80 participants. Chihuahua, Chih, Mexico, 2016

Variable	f^†^	%
Signs and symptoms		
Bleeding in digestive tract	47	58,8
Persistent abdominal pain	35	43,8
Diarrhea, constipation or both	40	50
Feeling of incomplete evacuation	22	27,5
Presence of blood in the stool	43	53,8
Pain in the digestive tract	36	45
Protruding abdomen	35	43,8
Tiredness or anemia	25	31,3
Unexplained weight loss	42	52,5
Age at diagnosis		
CRC* not related to age	37	46,3
Risk factors		
Consumption of one alcoholic beverage or more per day	21	26,3
Low intake of fruits and vegetables	28	35
Intake of red or processed meat daily	34	42,6
Low fiber diet	27	33,8
Being overweight	33	41,3
Being over 70 years of age	25	31,3
Having a close relative diagnosed with CRC*	40	50
Not performing physical activity	14	17,6
Having a bowel disease such as colitis	27	33,8
Having diabetes	15	18,8
Prevention		
Knowledge about prevention programs	36	45
Confidence		
Confidence in detecting signs and symptoms	11	13,8

*CRC = Colorectal Cancer; ^†^ *f =* frequency

The Pearson’s Correlation Coefficient test was performed to identify the relationship between the variables (age, alcohol use, tobacco smoking, food intake, BMI) and CRC awareness. No statistically significant association was found between the study variables and CRC risk awareness (*p*> 0.05) (Table 4).

**Table 4:** Correlation for the CRC* risk awareness of the 80 participants. Chihuahua, state of Chihuahua, Mexico, 2016

Variables	CRC* Risk Awareness	p^†^
Age	-0.056	0.647
Alcohol Use	0.023	0.850
Tobacco Smoking	0.013	0.914
Food Intake	0.057	0.640
BMI^‡^	-0.082	0.499

*CRC = Colorectal Cancer; †*p =* Pearson’s Correlation Coefficient Test; ‡BMI = Body Mass Index

The contingency coefficient test was performed to determine the CRC risk awareness by sex, and no statistically significant association was found between the variables (p = 0.483). Similarly, the marital status was considered and no statistically significant association was found (p = 0.274).

## Discussion

The results of this study confirm that changes in lifestyle and the adoption of unhealthy habits have led to a decrease in the timely prevention and detection of CRC. Therefore, there is a need for programs focused on these two measurements, and since the success of prevention programs depends on the active participation of the population, their dissemination plays an important role in their acceptance.

According to the evidence, being over 50 years represents a risk factor for developing CRC. However, when the LS is present, CRC can be diagnosed in people under 30 years of age, and the average age in this study was 36.8 years. A study conducted in Hungary showed that up to 32.7% of participants between 40 and 70 years of age who received guidance and indication to get a CRC screening test were more likely to undergo a physical examination. On the other hand, a lower knowledge of risk factors among people under 50 years of age has been reported, which is considered as one of the main reasons for not requesting a CRC screening due to the distress and discomfort caused by the procedure^32^.

A study conducted with a population from Australia showed that a family history of CRC increases the participation of people for a timely detection. Likewise, having knowledge about the disease and being married or in a relationship are more likely to participate in CRC screening programs^33^. The results of this research show that most of the participants are married and the most prevalent risk factor was having a close relative diagnosed with CRC, although only half of the participants knew it. However, no association between these factors and CRC awareness was found.

In this study, participants have a family history and known risk factors for the development of CRC (overweight, alcohol use and intake of meat and fat), and it is noted that most participants knew at least some of the signs and symptoms that can cause CRC, at the time of diagnosis. However, few of them identify the risk factors, and there is little knowledge about prevention programs.

It is necessary to increase the level of knowledge and strengthen the information in terms of prevention so that the population recognizes the risk factors, with emphasis on those that can be modified. This type of intervention is cost effective in public health, as well as a permanent campaign for the timely diagnosis of the disease, because even in screening programs there is a lack of CRC awareness, which makes the participation of the population in diagnostic studies to be around 70.3%^34^.

Alcohol use has been identified as one of the main risk factors for CRC, and almost half of the population in this study showed symptoms of alcohol dependence. A study conducted in Spain reports that primary care professionals, including nurses, find it difficult to identify patterns of alcohol use among the population, but it is necessary to overcome this obstacle to get to know the use of licit and illicit substances when family risk factors for CRC are present^35^. It is necessary to design and implement more effective and lasting strategies to increase knowledge about risk factors and CRC awareness. By this, prevention programs with a nationwide scope are designed and hence, the costs of medical care, complications, hospitalizations and associated death are reduced.

The objective of this study was to identify the association between environmental risk factors and CRC awareness, although the results found were not statistically significant. In addition, a low knowledge about the risk factors, signs and symptoms of CRC was observed, with these results being similar to those reported in another study^19^. Because it is a population at familial risk, there is a higher probability of developing the disease at an early age. Given that the nursing professional actively participates in health education, it is necessary that they can identify people at risk for LS and inform them about environmental factors with the aim of modifying unhealthy lifestyles.

The nursing professionals, due to their training in the biological and behavioral areas, are the best people to carry out studies to identify the population at risk of getting sick. Moreover, during their work in the clinical field, they can recognize the familial and hereditary risks, such as the LS, in people, which can reduce the exposure to factors associated to unhealthy lifestyles.

One of the limitations in this study was the small sample used, because several of the records in the institutions were incomplete and did not meet the initial selection criteria for LS. It was difficult to locate people at first, but after making the invitation and informing them about the benefits of the study, they agreed to participate.

## Conclusion

There is scientific evidence on the environmental and family risks for developing CRC. In this study, overweight and alcohol use are two of the most prevalent modifiable factors, and the low level of knowledge about the CRC risk factors, signs and symptoms results in a lack of CRC awareness and, therefore, an increased incidence of CRC is likely. No association between risk factors and CRC awareness was found in this study, but it is suggested to replicate it with a larger sample.

It is important that the nursing staff contribute to the education on CRC by informing people of the environmental and hereditary risk factors to prevent or early diagnose the disease.
